# Development and Validation of a Clinical Prognostic Model Based on Immune-Related Genes Expressed in Clear Cell Renal Cell Carcinoma

**DOI:** 10.3389/fonc.2020.01496

**Published:** 2020-08-28

**Authors:** Shiqi Ren, Wei Wang, Hanyu Shen, Chenlin Zhang, Haiyan Hao, Mengjing Sun, Yingjing Wang, Xiaojing Zhang, Bing Lu, Chen Chen, Ziheng Wang

**Affiliations:** ^1^Department of Clinical Biobank, Nantong University Affiliated Hospital, Nantong, China; ^2^Department of Medicine, Nantong University Xinling College, Nantong, China; ^3^Medical School of Nantong University, Nantong, China; ^4^Department of Orthopaedics, Qidong Hospital of Chinese Medicine, Nantong, China; ^5^Department of Urology, Affiliated Hospital of Nantong University, Nantong, China; ^6^Department of Pathology, Medical School of Nantong University, Nantong, China; ^7^Department of Oncology, Jiangsu Cancer Hospital and Jiangsu Institute of Cancer Research and Nanjing Medical University Affiliated Cancer Hospital, Nanjing, China

**Keywords:** clear cell renal cell carcinoma, TCGA, GEO, immune-related genes, clinical prognostic model, tumor microenvironment

## Abstract

**Background:** Clear cell renal cell carcinoma (ccRCC) is the most frequent and terminal subtype of RCC. Reliable markers associated with the immune response are not available to predict the prognosis of patients with ccRCC. We exploited the extensive number of ccRCC samples from The Cancer Genome Atlas (TCGA) and Gene Expression Omnibus (GEO) repository to perform a comprehensive analysis of immune-related genes (IRGs).

**Methods:** Based on TCGA data, we incorporated IRGs and their expression profiles of 72 normal and 539 ccRCC samples. Univariate Cox analysis was used to evaluate the relationship between overall survival (OS) and IRGs expression. The Lasso Cox regression model identified prognostic genes used to establish a clinical immune prognostic model. The TF–IRG network was used to study the potential molecular mechanisms of action and properties of ccRCC-specific IRGs. Multivariate Cox analysis established a clinical prognostic model of IRGs.

**Results:** We found a significant correlation among 15 differentially expressed IRGs with the OS of patients with ccRCC. Gene function enrichment analysis showed that these IRGs are significantly associated with response to receptor ligand activity. Lasso Cox regression analysis identified 10 genes with the greatest prognostic value. A clinical prognostic model based on six IRGs, which performed well for predicting prognosis, revealed significant associations of patients' survival with age, sex, stage, tumor, node, and metastasis. Moreover, these findings reflect the infiltration of tumors by various immune cells.

**Conclusion:** We identified six clinically significant IRGs and incorporated them into a clinical prognostic model with great significance for monitoring and predicting prognosis of ccRCC.

## Introduction

Renal cell carcinoma (RCC) is a frequent cause of mortality of patients with urinary cancer, accounting for 2% of malignant tumors of adults (1). Annually, there are ~350,000 new cases of RCC worldwide, leading to ≥140,000 annual fatalities (2). Clear cell renal cell carcinoma (ccRCC) is the most frequent and lethal subtype, accounting for 75% of RCCs (3). Although the treatment of ccRCC has significantly improved during the past 10 years, there are limitations to its diagnosis, treatment, and prognosis. Distant metastasis occurs in 30% of patients with ccRCC who undergo surgery during the early stages of disease (4). Further studies of the mechanisms of ccRCC occurrence and development are therefore required, as well as efforts to develop new diagnostic methods and to identify potential biomarkers.

The components of the tumor microenvironment, which contribute to the development of tumors, include immune cells, stromal cells, extracellular matrix molecules, cytokines, and chemokines (5). These components reflect the evolutionary nature of tumor progression, which promotes immune escape, tumor growth, and metastasis (6). Moreover, new therapeutic targets have been identified through studies of these components and their complex interactions (5). For example, Li et al. (7) investigated the prognostic value of immune-related genes (IRGs) to establish an individual's immune characteristics and to improve predictions of the prognosis of patients with non-small cell lung cancer (7). Thus, understanding the molecular and cellular composition and function of the ccRCC tumor microenvironment is required to improve prognosis and to identify new biomarkers (8, 9).

Publicly available gene expression datasets and the emergence of related platforms such as The Cancer Genome Atlas (TCGA) database provide readily accessible and convenient platforms for rapid and accurate identification of biomarkers for monitoring tumors (10, 11). For example, Yoshihara et al. (8) studied the tumor microenvironment by analyzing the expression of specific molecular biomarkers of immune and stromal cells using an estimation algorithm employing stromal and immune scores. Such estimation algorithms evaluate the prognosis of many tumors and identify biomarkers (8, 9, 12, 13). However, there is no definitive threshold to aid studies of the associations of clinical correlates and prognostic significance with the tumor microenvironment and ccRCC.

Here we aimed to comprehensively study the possible clinical efficacy of IRGs in the ccRCC tumor microenvironment to stratify prognosis, as well as their potential value as biomarkers for targeted therapy. For this purpose, we combined the expression profiles of IRGs with clinical information to evaluate overall survival (OS). We systematically analyzed the expression of ccRCC IRGs and their associations with prognosis to develop personalized prognostic markers. Furthermore, bioinformatics analysis was used to identify potential regulatory mechanisms. The results of this study will provide the basis for research related to immunization and provide a theoretical basis for the development of individualized therapy.

## Materials and Methods

### Data Collection and Clinical Samples

We acquired ccRCC transcriptomic sequencing data from TCGA data (https://portal.gdc.cancer.gov/), including 539 ccRCC and 72 normal samples. Patients' clinical information was extracted as well. Gene expression matrix files and clinical information from the GSE29609 dataset were obtained from the Gene Expression Omnibus (GEO) repository. The list of IRGs was exported from the immunology database and analysis portal (ImmPort) database that provides immunology data (14). Moreover, the database provides a list of IRGs associated with processes that mediate the immune response.

### Analysis of Differentially Expressed Genes

The edgeR package was used to screen IRGs differentially expressed between ccRCC and normal samples (15). Log_2_ transformation was used to standardize the raw data. We applied differential gene expression (DGE) analysis using cut-off values of |log_2_ fold change| > 1 and FDR <0.05. Then, we extracted the differentially expressed IRGs from all DEGs. The molecular mechanisms potentially responsible for the differential expression of IRGs were investigated using functional enrichment analysis of the GO and KEGG pathways (16–18) using the clusterProfiler package (19).

### Survival Analysis

Clinical information were acquired from TCGA data and the GEO database. To analyze OS, we used the R survival and survminer packages. We conducted single-variable Cox analysis using the R survival package to identify survival-related IRGs.

### Molecular Characteristics of Prognosis-Related IRGs

Analyses of the differential expression of IRGs related to the prognosis of patients with ccRCC may have clinical value. To investigate functional interactions, we constructed a protein–protein interaction (PPI) network using the STRING database (http://string-db.org) (20). PPI networks show direct or indirect interactions of gene products. Cytoscape was used to visualize the results of the PPI network (21). Moreover, transcription factors (TFs) directly control gene expression. We focused on potential target transcription factors (TFs) of these prognosis-related IRGs. To identify the regulatory links between the TFs and the transcriptome, we employed the Cistrome Cancer database (http://cistrome.org/), which incorporates TCGA data with >2,300 ChIP-seq data and analyses of chromatin accessibility. We constructed a regulatory network of potential TFs and current IRGs by considering TFs of clinical significance.

### Construction and Verification of a Prognostic Model

We used the Lasso method to select the main IRGs from the important cohort of the Cox univariate regression analysis, which identifies the subclass of IRGs associated with the prognosis with ccRCC. This was achieved by considering lowering the regression coefficient by suppressing the penalty score compared with its size. Finally, a few indicators with nonzero weights persisted, while those of most possible indicators approached zero. Therefore, the proportional hazards regression calculated using the Lasso method further reduced the representation of immune-related genes. We next generated a sample of an existing sample dataset using 1,000 iterations, selected IRGs repeated 900 times, and used the “glmnet” R package to complete the Lasso Cox analysis. Finally, we used β coefficients of multiple regression analysis to establish a prognostic immune correlation model. These coefficients were multiplied by the expression level of each immune-related gene.

### Clinical and Immune Correlations of the Prognostic Model

The classification of patients into high- and low-risk groups was performed according to their risk scores, and prognosis was evaluated. The TIMER database (https://cistrome.shinyapps.io/timer/) analyzes and visualizes the abundance of tumor-infiltrating immune cells (22). Here we analyzed these data for patients with ccRCC and calculated their correlations with IRGs to establish a model of clinical prognosis and immune cell infiltration.

### Statistical Analysis

We identified the functions of the prognostic features using the survivalROC R package to calculate survival according to the area under the curve (AUC) of the receiver operator characteristic (ROC) curve (23). Significant and acceptable predictive values were defined as AUC ≥ 0.75 and AUC ≥ 0.6, respectively. Statistical analysis was performed using R software, and *P* < 0.05 indicates a significant difference.

## Results

### Identification of Differentially Expressed IRGs

**Figure 1:**
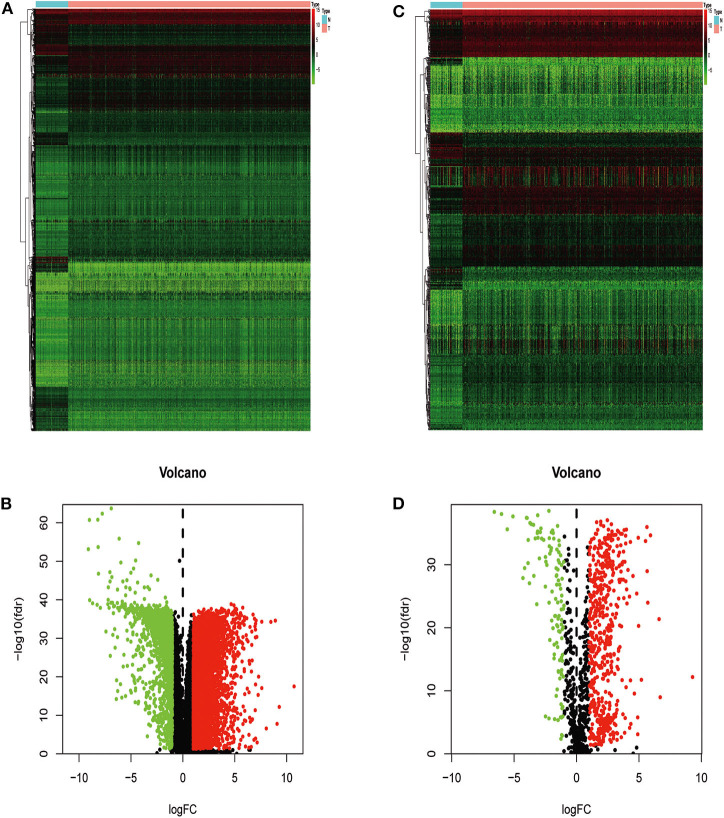
Differentially expressed immune-related genes. Heatmap **(A)** and volcano plot **(B)** showing genes differentially expressed between clear cell renal cell carcinoma (ccRCC) and normal tissues. Green, red, and black dots represent genes expressed at relatively lower, higher, or equal levels. The differentially expressed immune-related genes (IRGs) are shown in a heatmap **(C)** and volcano plot **(D)**. Green, red, and black dots represent genes expressed at relatively lower, higher, or equal levels.

We extracted 7,369 genes and 611 samples from TCGA ccRCC data, including 1,902 upregulated genes and 5,467 downregulated genes (Figures 1A,B). We extracted 681 differentially expressed IRGs from this set of genes, which included 116 downregulated and 565 upregulated genes (Figures 1C,D). Gene function enrichment analysis showed that the immune response-regulating cell surface receptor signaling pathway, the external side of the plasma membrane, and antigen binding were the most common biological terms among biological processes, cell components, and molecular functions, respectively (Table 1). Furthermore, KEGG pathway analysis revealed that these IRGs (Table 2) are significantly involved in cytokine–cytokine receptor interactions, viral protein interactions with cytokines, and natural killer cell-mediated cytotoxicity. Table 3 showed the first reported IRGs in ccRCC.

**Table 1:** Gene function enrichment of differentially expressed immune related genes.

**Ontology**	**ID**	**Description**	***p*.adjust**	**Count**
BP	GO:0002460	Adaptive immune response based on somatic recombination of immune receptors built from immunoglobulin superfamily domains	3.78E-106	138
BP	GO:0002449	Lymphocyte mediated immunity	8.76E-106	136
BP	GO:0002429	Immune response-activating cell surface receptor signaling pathway	1.69E-94	137
BP	GO:0002768	Immune response-regulating cell surface receptor signaling pathway	1.76E-93	140
BP	GO:0016064	Immunoglobulin mediated immune response	1.76E-93	105
BP	GO:0019724	B cell mediated immunity	2.81E-93	105
BP	GO:0006959	Humoral immune response	3.63E-92	126
BP	GO:0002455	Humoral immune response mediated by circulating immunoglobulin	3.63E-92	91
BP	GO:0006958	Complement activation, classical pathway	3.20E-90	87
BP	GO:0050900	Leukocyte migration	1.36E-85	137
CC	GO:0009897	External side of plasma membrane	1.98E-83	118
CC	GO:0042571	Immunoglobulin complex, circulating	2.96E-59	52
CC	GO:0019814	Immunoglobulin complex	2.99E-59	53
CC	GO:0042611	MHC protein complex	4.97E-26	21
CC	GO:0043235	Receptor complex	1.18E-24	64
CC	GO:0072562	Blood microparticle	7.47E-21	37
CC	GO:0071556	Integral component of lumenal side of endoplasmic reticulum membrane	7.61E-17	17
CC	GO:0098553	Lumenal side of endoplasmic reticulum membrane	7.61E-17	17
CC	GO:0042613	MHC class II protein complex	8.96E-16	13
CC	GO:0012507	ER to Golgi transport vesicle membrane	2.60E-11	18
MF	GO:0003823	Antigen binding	9.43E-163	140
MF	GO:0048018	Receptor ligand activity	7.44E-75	124
MF	GO:0034987	Immunoglobulin receptor binding	2.39E-57	52
MF	GO:0005125	Cytokine activity	6.65E-54	75
MF	GO:0005126	Cytokine receptor binding	3.96E-45	75
MF	GO:0004896	Cytokine receptor activity	6.41E-36	42
MF	GO:0004252	Serine-type endopeptidase activity	4.44E-35	62
MF	GO:0008236	Serine-type peptidase activity	9.84E-33	62
MF	GO:0017171	Serine hydrolase activity	2.15E-32	62
MF	GO:0008083	Growth factor activity	2.70E-29	47

**Table 2:** The top 10 most significant Kyoto Encyclopedia of Genes and Genomes pathways (KEGG).

**ID**	**Description**	**P.adjust**	**Count**
hsa04060	Cytokine-cytokine receptor interaction	1.88E-67	117
hsa04061	Viral protein interaction with cytokine and cytokine receptor	1.41E-35	52
hsa04650	Natural killer cell mediated cytotoxicity	2.82E-25	49
hsa04612	Antigen processing and presentation	4.09E-21	35
hsa04640	Hematopoietic cell lineage	4.17E-18	36
hsa04658	Th1 and Th2 cell differentiation	1.93E-16	33
hsa04062	Chemokine signaling pathway	1.97E-15	46
hsa04659	Th17 cell differentiation	3.35E-15	34
hsa04514	Cell adhesion molecules (CAMs)	5.70E-13	37
hsa04630	JAK-STAT signaling pathway	1.31E-11	37

**Table 3:** First reported immune microenvironment- related genes in ccRCC.

**Gene symbol**	**logFC**	***p*-value**	**FDR**
AEN	1.39002	<0.001	<0.001
ANGPTL7	−1.06387	<0.001	<0.001
APLN	2.486788	<0.001	<0.001
AZGP1	−1.75511	<0.001	<0.001
BLNK	−1.15577	<0.001	<0.001
BMP5	−1.27325	<0.001	<0.001
BMP8A	1.442843	<0.001	<0.001
C3AR1	1.909827	<0.001	<0.001
CARD11	2.086722	<0.001	<0.001
CKLF	1.085822	<0.001	<0.001
CSF3R	2.789211	<0.001	<0.001
EBI3	2.313785	<0.001	<0.001
FAM3B	−4.0026	<0.001	<0.001
FCGR2B	2.001925	<0.001	<0.001
FPR1	1.584903	<0.001	<0.001
HCST	1.980888	<0.001	<0.001
HSPA6	2.069337	<0.001	<0.001
IGHA2	2.137115	<0.001	<0.001
IGHJ2	2.46991	<0.001	<0.001
IL2RA	2.276302	<0.001	<0.001
INPP5D	1.711738	<0.001	<0.001
PPARA	−1.00738	<0.001	<0.001
RAET1E	−1.80059	<0.001	<0.001
TNFSF14	3.76326	<0.001	<0.001

*logFC, log fold change (tumor tissues vs. normal tissues); FDR, false discovery rate*.

### Identification of Prognosis-Related IRGs

**Figure 2:**
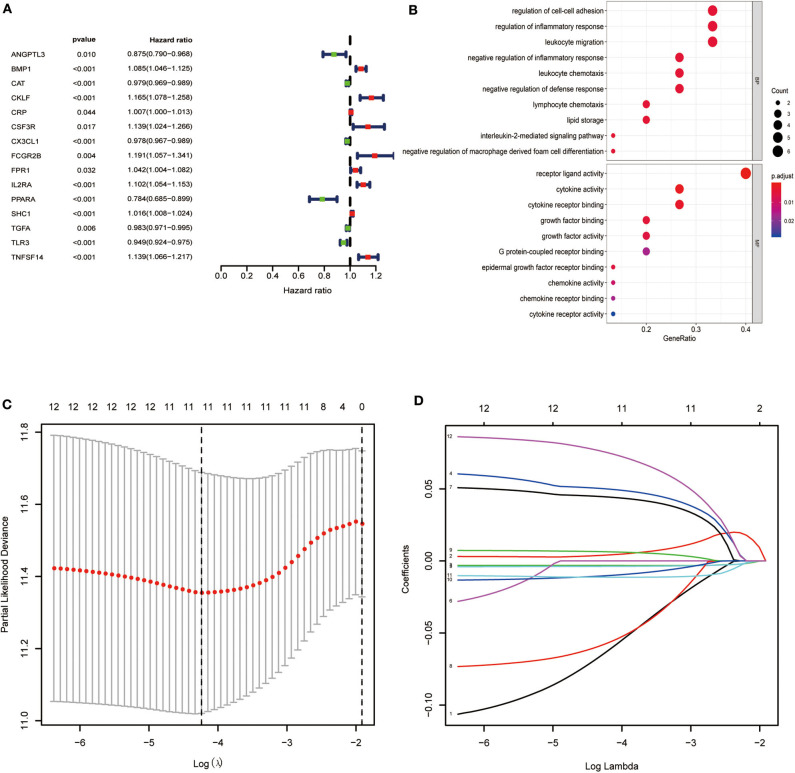
Identification of immune-related genes with the most significant prognostic value. **(A)** Forest plot of hazard ratios showing the prognostic value of survival-related immune-related genes. **(B)** Gene function enrichment (GO) analysis of survival-related immune-related genes. **(C,D)** The Lasso regression method based on the glmnet package was used to identify the 21 most prognostic IRGs in TCGA training group.

We found a significant association of 15 IRGs with OS. A forest hazard map shows that most of these IRGs serve as risk factors for ccRCC (Figure 2A), and gene function enrichment analysis revealed that these IRGs are significantly associated with response to receptor ligand activity (Figure 2B). Furthermore, Lasso Cox regression analysis identified 10 genes with the highest prognostic values (Figures 2C,D).

### A Gene Regulatory Network Comprising TFs and IRGs

**Figure 3:**
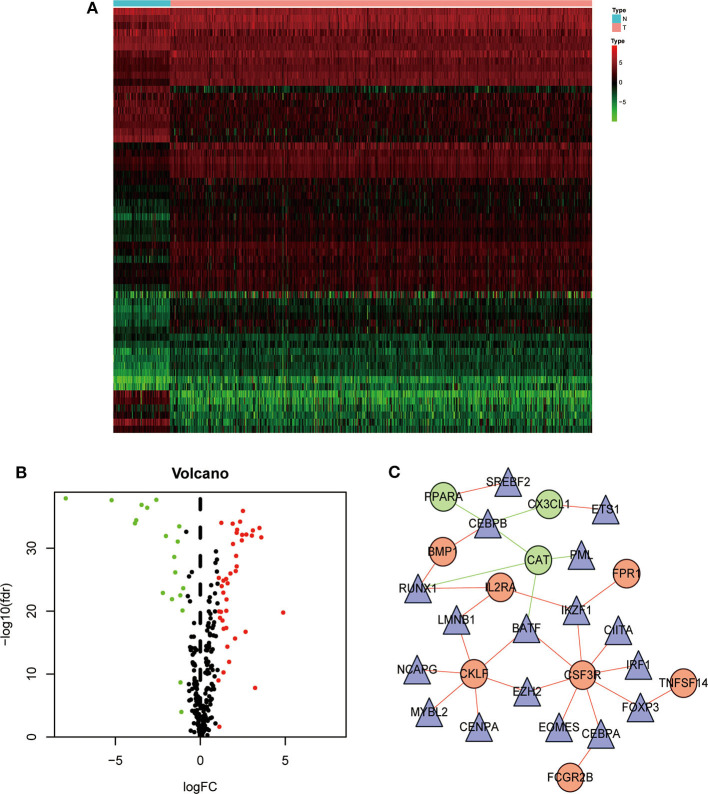
Construction of a TF–immune-related gene regulatory network. Differentially expressed transcription factors (TFs) are shown in a heatmap **(A)** and a volcano plot **(B)**. Green, red, and black dots represent genes expressed at relatively lower, higher, or equal levels. **(C)** A regulatory network comprising TFs and IRGs. Triangles represent TFs, and red and green indicate risk and protective factors, respectively.

We next analyzed the regulatory mechanisms of TF genes and IRGs to identify the molecular mechanisms linked to their clinical significance. When we analyzed the expression profiles of 318 TFs, we identified 60 differentially expressed TFs (Figures 3A,B). A regulatory network constructed using these 60 TFs and 15 IRGs. The critical values were correlation coefficient = 0.4 and *P* = 0.6. The resulting TF-based regulatory networks clearly illustrated the regulatory relationships between these IRGs (Figure 3C).

### Development of a Clinical Prognostic Model

Here we identified six IRGs according to the results of the Lasso Cox model analysis, which were used to develop a prognostic model of the IRGs, *ANGPTL3, IL2RA, PPARA, SHC1, TGFA*, and *TNFSF14* (Table 4). The risk score was calculated as follows: [expression level *ANGPTL3* ^*^ (−0.1200)] + [expression level *IL2RA* ^*^ (0.0577)] + [expression level *PPARA* ^*^ (−0.1445)] + [expression level *SHC1* ^*^ (0.0105)] + [expression level *TGFA* ^*^ (−0.0159)] + [expression level *TNFSF14* ^*^ (0.1075)].

**Table 4:** Information on IRGs used to construct clinical prognostic models.

**IRGs**	**Coef**	**HR**	***p-*value**
ANGPTL3	−0.1200	0.8870	0.0209
IL2RA	0.0577	1.0594	0.0401
PPARA	−0.1445	0.8655	0.0431
SHC1	0.0105	1.0106	0.0492
TGFA	−0.0159	0.9843	0.0152
TNFSF14	0.1075	1.1135	0.0046

*IRGs, immune-related genes; Coef, Cox-PH coefficient; HR, Hazard Ratio*.

### Evaluation of the Prognostic Performance of the Clinical Prognostic Model Based on IRGs

**Figure 4:**
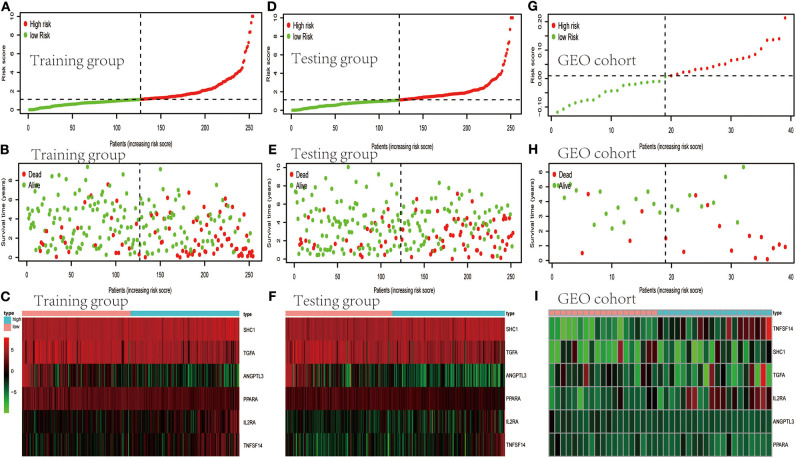
Evaluation and validation of clinical prognostic models employing IRGs. **(A,D,G)** Distribution of patients according to risk index. **(B,E,H)** Survival. **(C,F,I)** Heatmaps of IRG expression profiles.

**Figure 5:**
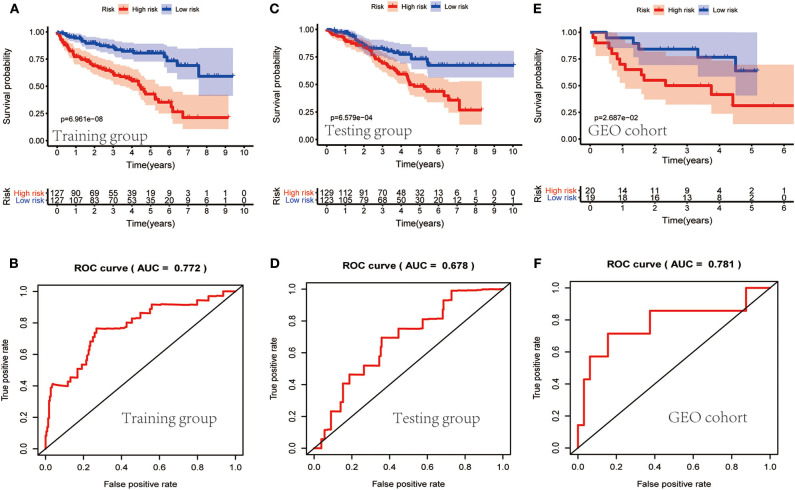
Evaluation of the clinical prognostic model. **(A,C,E)** The risk scores of the clinical prognostic model predict survival. **(B,D,F)** The receiver operating characteristic (ROC) curve of survival-dependent receiver verifies the prognostic value of the model.

**Figure 6:**
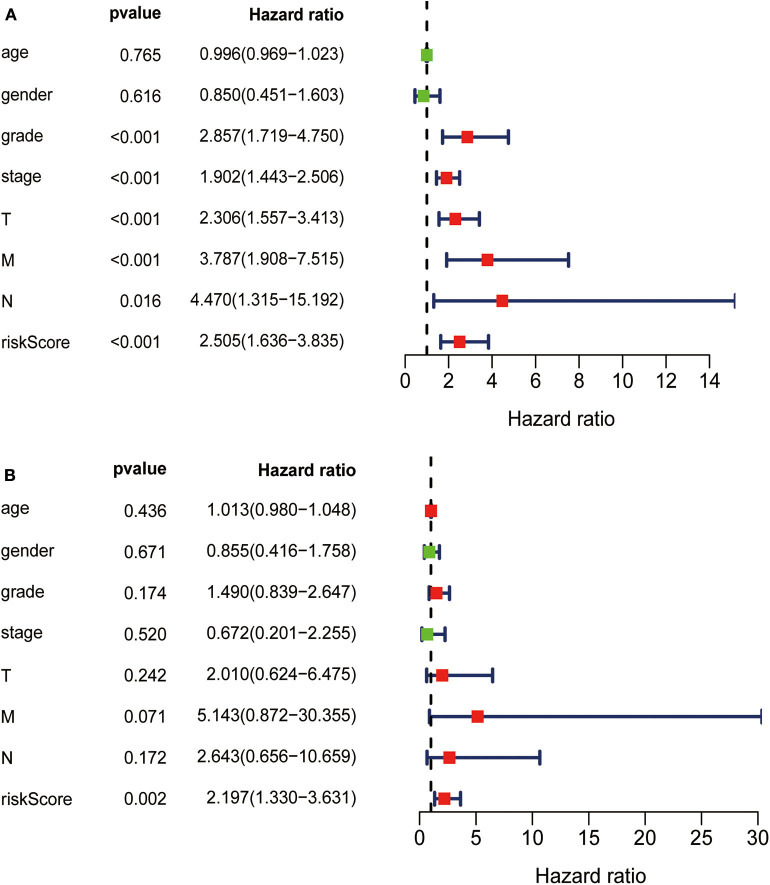
**(A)** Univariate analysis revealed that the risk score correlated with shorter OS, stage, grade, and TNM. **(B)** Multivariate analysis revealed that the risk score served as an independent prognostic factor.

TCGA clinical data of 504 patients with ccRCC included age, sex, stage, tumor, node, metastasis stage, and survival. These patients were randomly divided into a training (*n* = 252) or test (*n* = 252) group. Table 5 shows their clinical information. According to the risk scores of the prognostic model, patients with ccRCC were divided into a low- or high-risk group (Figure 4A). As the risk score increased, the longevity of patients decreased (Figure 4B). Figure 4C shows differential expression of the IRGs between the low- and high-risk groups. The clinical prognostic model yielded a risk score that predicted that the OS rates of the low- and high-risk groups were significantly different (Figure 5A). The AUC of the ROC curve was 0.772, indicating that the prognostic features based on IRGs were highly accurate for predicting survival (Figure 5B). Furthermore, univariate analysis revealed that the risk score significantly correlated with shorter OS (HR: 2.50; 95% CI: 1.64–3.83; *P* < 0.001). Other clinicopathologic variables associated with poor survival included stage, and grade as well as tumor, node, and metastasis stage. Multivariate analysis indicated that the risk score served as an independent prognostic factor (HR: 2.20; CI: 1.33–3.63, *P* = 0.002) (Figures 6A,B, Table 6).

**Table 5:** Clinical characteristics of ccRCC patients included in this study.

**Variables**	**Total TCGA-KIRC**	**Training group**	**Testing group**	**GEO cohort**
	**(*****N*** **=** **504)**	**(*****N*** **=** **252)**	**(*****N*** **=** **252)**	**(*****N*** **=** **39)**
Age (Mean ± SD)	60.47 ± 12.16	61.71 ± 11.82	59.24 ± 12.39	61.38 ± 12.77
Survival time (y)	3.27 ± 2.18	3.13 ± 2.21	3.40 ± 2.15	2.99 ± 1.67
Status				
Alive	339 (67.26)	169 (67.06)	170 (67.46)	22 (56.41)
Dead	165 (32.74)	83 (32.93)	82 (32.54)	17 (43.59)
Gender				
Male	331 (65.67)	157 (62.30)	174 (69.05)	
Female	173 (34.33)	95 (37.70)	78 (31.95)	
Stage				
I	249 (49.70)	123 (49.00)	126 (50.40)	
II	53 (10.57)	30 (11.95)	23 (9.20)	
III	117 (23.35)	58 (23.11)	59 (23.60)	
IV	82 (16.37)	40 (15.94)	42 (16.80)	
Grade				
1	10 (2.01)	3 (1.21)	7 (2.80)	
2	215 (43.26)	110 (44.53)	105 (42.00)	
3	198 (39.84)	103 (41.70)	95 (38.00)	
4	74 (14.89)	31 (12.55)	43 (17.20)	
*T*				
1	255 (50.60)	127 (50.40)	128 (50.79)	11 (28.21)
2	65 (12.90)	36 (14.29)	29 (11.51)	5 (22.73)
3	173 (34.33)	82 (32.54)	91 (36.11)	22 (56.1)
4	11 (2.18)	7 (2.78)	4 (1.59)	1 (2.56)
*M*				
0	400 (83.68)	201 (84.45)	199 (82.92)	26 (66.67)
1	78 (16.32)	37 (15.55)	41 (17.08)	13 (33.33)
*N*				
0	224 (93.33)	112 (93.33)	112 (93.33)	32 (82.05)
1	16 (6.67)	8 (6.67)	8 (6.67)	7 (17.95)

*Data are shown as n (%). T, tumor; M, metastasis; N, node*.

**Table 6:** Univariate analysis and multivariate analysis of the correlation between the risk score calculated by the clinical prognosis model and OS.

	**Univariate analysis**		**Multivariate analysis**	
**Clinicopathologic**	**HR (95%CI)**	***p*-value**	**HR (95%CI)**	***p*-value**
Age	1.00 (0.97–1.02)	0.765		
Gender	0.85 (0.45–1.60)	0.616		
Grade	2.86 (1.72–4.75)	<0.001	1.49 (0.84–2.65)	0.174
Stage	1.90 (1.44–2.51)	<0.001	0.67 (0.20–2.25)	0.520
T	2.31 (1.56–3.41)	<0.001	2.01 (0.62–6.47)	0.242
M	3.79 (1.91–7.52)	<0.001	5.14 (0.872–30.35)	0.071
N	4.47 (1.31–15.19)	0.016	2.64 (0.66–10.66)	0.712
Risk score	2.50 (1.64–3.83)	<0.001	2.20 (1.33–3.63)	0.002

*Bold values indicate P <0.05. HR, hazard ratio; CI, confidence interval. T, Tumor; N, Node; M, Metastasis*.

### Validation of the Clinical Prognostic Model

To determine whether the clinical prognostic model was reliable when applied to different populations, we used the same formula to evaluate the test group and the GEO cohort (GSE29609), which was consistent with the results of the training group. The GSE29609 data include 39 patients with ccRCC (Table 5). Patients were divided into high- or low-risk groups according to the risk value of the model (Figures 4D,G). Increased risk was associated with more deaths (Figures 4E,H). The results show further that the prediction potential of the clinical prognostic model was suitable for different populations. Figures 4F,I show the expression data of selected IRGs for different risk groups. Furthermore, the probability of survival of the high-risk group was lower than that of the low-risk group (Figures 5C,E). Next, we evaluated the accuracies of the clinical prognostic model applied to the test group and GEO cohort, for which the AUCs of the ROC curve were 0.678 and 0.781, respectively (Figures 5D,F). These results indicate that the clinical prognostic model accurately predicted the prognosis of patients with ccRCC.

### Clinical and Immune Correlations of the Prognostic Model

**Figure 7:**
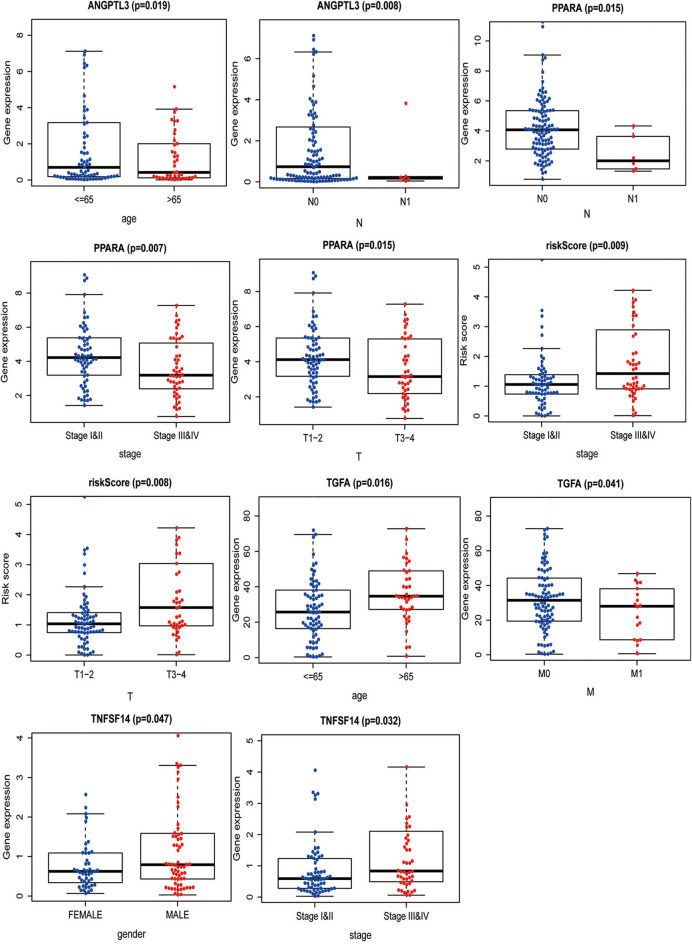
Correlation between IRGs and patients' clinical and demographic characteristics.

**Figure 8:**
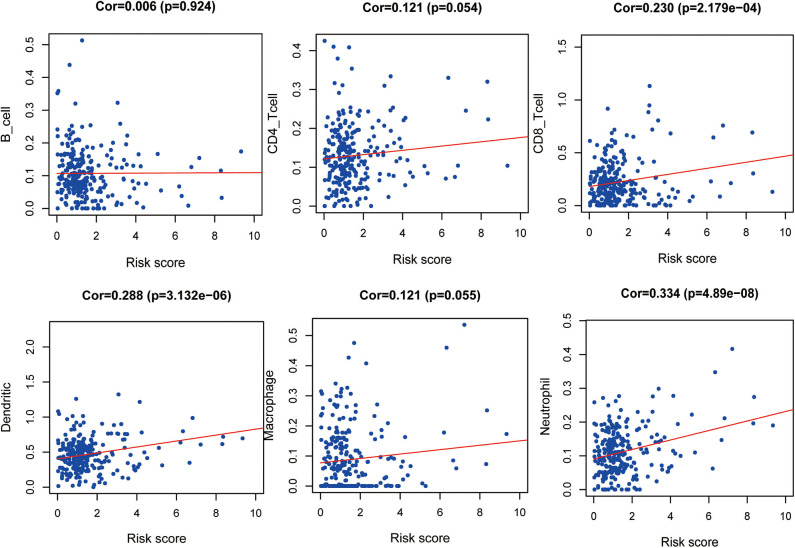
Relationship between risk score and immune cell infiltration.

The correlation between the IRGs analyzed using the clinical prognostic model with clinical and demographic characteristics was analyzed as a function of age, sex, stage, and TNM stage (Figure 7). Furthermore, to determine whether the immune prognostic model accurately reflected the state of the tumor immune microenvironment, we analyzed the relationship between risk scores and immune cell infiltration. The results show that the risk score was significantly related to CD8^+^T cells (*p* < 0.001), neutrophils (*p* < 0.001), and dendritic cells (*p* < 0.001) (Figure 8).

## Discussion

The role of IRGs in tumorigenesis and development is established. However, systematic, comprehensive data that identify their roles in patients with ccRCC are insufficient. To address this deficiency in our knowledge, here we analyzed the ccRCC dataset of TCGA to establish a clinical prognostic model employing differentially expressed IRGs that accurately predicted the clinical outcomes of patients according to their clinicopathological characteristics. Moreover, these IRGs are closely associated with the occurrence and development of ccRCC and therefore may serve as significant clinical biomarkers. These results show that our clinical prognostic model predicted patients' outcomes as well as identified potential targets of immunotherapy.

Specifically, we identified 15 IRGs closely related to the survival of patients, including six protective factors and nine risk factors. Functional enrichment analysis showed that these IRGs are significantly associated with response to receptor ligand activity. To improve the accuracy of the clinical prognostic model, we used the Lasso Cox regression model to identify IRGs with the greatest prognostic value. Moreover, to study the molecular mechanisms that explain the possible clinical value of these IRGs, we established a TF-mediated network that considered significant differentially expressed TFs regulated by these IRGs. The regulatory network contained 17 TFs and 10 IRGs. Our TF–IRG regulatory network will provide guidance for future mechanistic analyses.

The present clinical prognostic model comprised six IRGs with prognostic significance. For example, angiopoietin-like proteins (ANGPTLs) (24) mediate lipid metabolism, inflammation, cancer cell infiltration, and hematopoietic stem cell expansion (24–28). Low levels of ANGPTL3 in RCC tissue are associated with poor prognosis (29), and ANGPTL3 inhibits metastasis of RCC by regulating the activities of MMPs and epithelial-mesenchymal transition (EMT)-related pathways (29). SHC1 is expressed at higher levels in RCC tissues compared with normal tissues, suggesting its requirement for the progression of ccRCC (30). SHC1 regulates PTRF through the AKT pathway to contribute to the occurrence and development of ccRCC (30).

Signaling through NF-κB-mediate pathways promotes tumor cell proliferation, inhibits apoptosis, induces angiogenesis and the EMT, and promotes distant metastasis. The activation of NF-κB may reshape local metabolism and energize the immune system, thereby promoting tumor growth (31, 32). TNFSF14 induces the noncanonical NF-κB pathway in certain types of cancer cells to promote tumor development (33). The nuclear transcription factor peroxisome proliferator-activated receptor-α (PPARA), a key mediator of lipid metabolism, serves as a biomarker for ccRCC (34). The high levels of IL2RA in activated circulating immune cells and Tregs is exploited for IL-2 immunotherapy of tumors and autoimmune diseases; and certain polymorphisms of *IL2RA* are related to the risk of kidney cancer (35, 36). Thus, these IRGs therefore provide a new direction for our research.

To evaluate the prognostic value of our clinical prognostic model, we determined the OS of patients with ccRCC in the training group. The prognostic model classified these patients into high- or low-risk groups for shorter survival according to risk scores. Moreover, when we generated risk curves by combining the changes in levels of six IRGs with clinical parameters, and by combining the risk scores of the prognostic model, we were able to monitor the progression of ccRCC. ROC curves indicated that high accuracy of the clinical prognostic model. All results were verified using a testing group and the GEO cohort. Multivariate analysis further confirmed that the risk score served as an independent predictor of OS of patients with ccRCC. Moreover, the prognostic model predicted the survival of patients as well as disease progression. Thus, this model will likely serve as a valuable tool to evaluate the prognosis of patients with ccRCC.

Moreover, our clinical prognostic model showed good clinical feasibility. For example, the six IRGs performed moderately for predicting prognosis and were associated with age, sex, grade, stage, and TNM stage. To analyze tumor–immune interactions, it is essential to characterize immune infiltration. Our analysis shows that the levels of the six IRGs positively correlated with the infiltration of neutrophils, dendritic, and CD8+ T cells. The role of neutrophils in cancer is multifactorial, and they participate in different stages of cancer development, including occurrence, growth, proliferation, and metastasis (37, 38). Furthermore, neutrophils promote tumor proliferation by weakening the immune system (39). Dendritic cells are required for the immune response through attracting antitumor T cells in the TME. However, during tumor development, dendritic cells may convert from immunostimulators to immunosuppressors (40). These results suggest that high-risk patients harbor relatively higher numbers of infiltrating dendritic cells, CD8+ T cells, and neutrophils. Moreover, our results suggest that these six IRGs may predict increased immune cell infiltration.

Previously, Ghatalia et al. and Wang et al. reported the ccRCC immune model, but the research of Ghatalia et al. was mainly based on ccRCC patients who received nephrectomy, and discussed the relationship between the characteristics of tumor infiltrating immune cells and the recurrence rate of local renal cancer (41). The difference is that our study is based on ccRCC patients and established a clinical immune gene model to predict the clinical prognosis of ccRCC patients. Another analysis of TGCA RCC data identified a prognostic 6-DEG classifier, including genes encoding IL21R, ATP6V1C2, GBP1, P2RY10, GBP4, and TNNC2 (42). Further analysis using this model revealed significant associations between immune/stromal scores and clinicopathological staging. The expression patterns of these genes expressed in the tumor microenvironment provide a powerful indicator of prognosis of patients with RCC. The differences in predictive IRGs identified by Wang et al. (42) Our present study may be explained by the former's use of the ESTIMATE package of R to score the immune/stromal of TCGA samples and then to screen differentially expressed genes using the Lasso Cox regression model to build a prognostic six gene-based clinical model to predict the survival of patients with ccRCC. In contrast, here we screened for differentially expressed IRGs acquired from the ImmPort database, and we then identified IRGs related to survival among the differentially expressed genes and used the Lasso Cox regression model to select IRGs with the highest ability to predict prognosis to construct the prognostic model. Furthermore, our prognostic model was validated using TCGA and GEO data, which yielded consistent, stable, and universal results.

In conclusion, our study identified and validated a clinical prognostic model comprising six IRGs, which served as an independent prognostic factor for patients with ccRCC. Moreover, the prognostic significance of this model may contribute to monitoring ccRCC occurrence and to predict prognosis. Our results provide new insights into approaches to develop new immunotherapies for ccRCC.

## Data Availability Statement

Publicly available datasets were analyzed in this study. This data can be found in The Cancer Genome Atlas (TCGA) database (https://portal.gdc.cancer.gov/) and Gene Expression Omnibus (GEO) database (https://www.ncbi.nlm.nih.gov/geo/).

## Author Contributions

SR, WW, and HS wrote the manuscript. CZ conducted bioinformatics analyses. MS and YW collected and processed data. XZ and HH prepared figures and tables. BL prepared the literature search and the bibliography, and references. ZW designed the article. CC reviewed the final draft of the manuscript.

## Conflict of Interest

The authors declare that the research was conducted in the absence of any commercial or financial relationships that could be construed as a potential conflict of interest.
